# Supplementary material for: Anatomical and biomechanical traits of broiler chickens across ontogeny. Part I. Anatomy of the musculoskeletal respiratory apparatus and changes in organ size
Source: PeerJ. 2014 Jul 3;2:e432. doi: 10.7717/peerj.432 (PMC4103091; doi:10.7717/peerj.432)
Supplement: Appendix — Results of the Shapiro-Wilk test to assess the normality of morphological data. [file peerj-02-432-s001.docx]

|  |  |  |  |  |  |  |  |  |  |  |  |
| --- | --- | --- | --- | --- | --- | --- | --- | --- | --- | --- | --- |
| **Age Group** | **Shapiro-Wilk test** | | | | | | | | | | |
|  | Body mass | Heart | Lung | Liver | Intestine | Wings | Girth | Keel length | Rib cage | Pectoralis Major | Pectoralis Minor |
| 1 day | 0.651 | 0.692 | 0.883 | 0.745 | 0.549 | n/a | n/a | n/a | n/a | 0.960 | 0.212 |
| 14 days | 0.989 | 0.308 | 0.917 | 0.178 | 0.691 | 0.496 | 0.219 | 0.711 | 0.799 | 0.104 | 0.686 |
| 28 days | 0.645 | 0.311 | 0.272 | 0.377 | 0.665 | 0.686 | 0.242 | 0.718 | 0.275 | 0.696 | 0.849 |
| 42 days | 0.765 | 0.340 | 0.216 | 0.125 | 0.225 | 0.668 | 0.716 | 0.153 | 0.213 | 0.410 | 0.229 |

Table 1: Results of Shapiro-Wilk tests to establish the normality of the data at each developmental stage.
